# Medical Jurisprudence

**Published:** 1824-06-01

**Authors:** 


### Medical Jurisprudence.

1. *Leach* versus *Veitch.* It appears that Dr. Leach, late of the British Museum, had been afflicted with insanity, and placed under the care of Dr. Veitch, a regular physician, who, during the late war, had charge, under government, of naval lunatics at Hoxton and elsewhere.

He was therefore a person properly qualified to treat Dr. Leach, till proved otherwise. It appears, however, that during medical treatment, the patient, (who was kept in a cottage out of town) and some of his friends, conceived that unnecessary severity was exercised by Dr. Veitch's keeper, and improper remedies exhibited by himself. The patient was therefore removed from Dr. V's care, and an action was brought against him for the recovery of damages, on the abovementioned two counts.

As far as we can judge from the evidence as stated in the Medical Repository, for February last, neither of these charges was proved. The charge of cruelty was chiefly maintained by an *old woman* who could not possibly have seen any of the transactions which she came forward to prove, and some discharged, and consequently malcontent servants, whose motives were more than questionable—or rather they were unquestionably bad. On this point, however, we shall not dwell, as Dr. Veitch's personal character was not involved, but only his *responsibility* for the acts of his servant.

In respect to the medical evidence brought forward to substantiate the charge of improper medical treatment on the part of Dr. Veitch, we must confess it does not altogether coincide with that liberality which we think one medical man should observe towards another, when called upon as a judge of actions and conduct which he did not witness, and therefore ought not to condemn, without knowing all the circumstances of the case, and all the indications which presented themselves to the former medical attendant. We shall give the two medical evidences, as stated in our cotemporary, leaving our readers to judge for themselves as to their ethical bearings. The first witness was Dr. Powell.

"He stated that when he saw Dr. Leach in July, 1822, soon after his removal from Dr. Veitch's, he found him in such a paralytic state as follows the use of mercury, and in a state of salivation; he thought him in extreme danger; he gave him *all the support he could* by nourishment and medicine. A *large* quantity of mercury would have produced the effect described, or a small quantity even on some constitutions. Being cross-examined, he admitted that he could not tell what had produced that state of the plaintiff's body—that exposure to cold, where no improper treatment had taken place, would produce the same effect. He further allowed, that if a patient were unwell in his body at the time he wanted to remove a mental malady, he would begin by eradicating the bodily disease; and if there was a liver complaint, he would take care to cure that before he made any other efforts." 173.

We really were not aware that *paralysis* is a usual sequence or consequence of the internal use of mercury, whether exhibited for hepatitis (which was the case here) syphilis, or insanity. If Dr. Powell will please to refer to Dr. Burnett's paper in the Philosophical Transactions, and also in the last Number of this Journal, he will find as broad a scale of mercurialization as he can possibly desire, on board His Majesty's ship Triumph, of 74 guns, whose crew were salivated in the extreme—some of whom died of the salivation; and where even the sheep, pigs, goats, and poultry were mercurialized; yet no instance is related of paralysis following, as a consequence of salivation. So again, in India, where hundreds and thousands are annually salivated in hepatitis, dysentery, &c. we rarely or never see paralysis ensue. If, in certain trades, where men are long exposed to noxious mercurial vapours, we have paralytic affections, will any man of candour consider such cases analogous to that of a common administration of mercury in a hepatic affection? We cannot, then, but regard Dr. Powell's coupling paralysis with mercury, in the above evidence, as uncalled for in an ethical, and incorrect in a pathological point of view. Dr. Powell must be well aware that insanity is generally (we might say invariably) the consequence of *corporeal disease*, especially of the *brain*. Is not paralysis therefore more likely to result from this state of the sensorium than from mercury? We leave this question to the judgement of the profession at large.

After the *old woman* and the discarded servant, *Capper*, had given their evidence as to the *mala praxis* of Dr. Veitch, Dr. Sutherland was called forward.

"Then came Dr. Sutherland, who saw the plaintiff about the 14th June, 1822, in a very weak state of health, apparently proceeding from the extremely frequent use of mercury. He changed the mode of treatment, and gave strengthening medicines. He considered the mode of treatment, *described by the last witness*, as most improper; but, being cross-examined, he admitted that he did not know the occasions on which the mercury was used, and could not therefore judge whether it was used improperly or not. The effect might have been produced on some constitutions even when used cautiously; and a short walk was very proper for persons in that state of health. Opening medicines were also necessary sometimes in these cases. Dr. Sutherland allowed Dr. Veitch to be a regular Physician." 173.

In this, as in the former evidence, we see the same apparent want of delicate feeling at the beginning—and the same admission of exceptions, enough to eat up the evidence in chief, on cross examination.

Our remarks are grounded, of course, on the *litera scripta* before us. If Dr. Powell and Dr. Sutherland considered the report of the case in the Repository as incorrect, it was their place to contradict it. Not having heard of any such contradiction, we are bound to believe the statement to be true. We shall now give the evidence of Mr. Wardrop.

"Mr. Wardrop had known the defendant about eighteen years, who has applied himself to the care of lunatic patients about ten years. When he saw Dr. Leach first, he was greatly irritated, and suffered under a disorder in the liver; and in complaints of that nature mercury is frequently employed. He thought mercury fit for the case of Dr. Leach; and it produced a gradual amendment. He left Dr. V.'s care quite sane. In five or six months he returned to the cottage. Witness there attended him professionally, and observed the same symptoms as on the former occasion. Being consulted as to the use of mercury, *he recommended it to be employed with considerable vigour.* At this time the action of the liver was considerably deranged, and the mode of treatment adopted by the defendent was very judicious." 174.

The judge, in summing up, observed, that there was nothing imputable to Dr. Veitch himself, but only to his servant—thus completely absolving him from the charge of *mala praxis* brought forward by non-professional gossips, and, with shame and sorrow be it spoken, *countenanced* by the medical witnesses, who ought to have been the guardians of their brother practitioner's reputation! There were damages of 50 pounds awarded for "improper coercion."

An application was made for a new trial, with the view of falsifying the statements of the principal evidence, Capper; but from some legal informality the application failed. We shall conclude this article with one or two extracts from our respected cotemporary.

"Thirdly, The jury came to a decision, with which we cannot meddle—for that is matter of *law*—but as we shall take the liberty of meddling with the *physic* of the matter, we are disposed to say, that we cannot comprehend upon what evidence that decision was founded. We shall not suppose that the learned housemaid was their Pallas: perhaps it was Dr. Powell. This gentleman, however, did not see the patient until some days after he was removed from Dr. Veitch's care. At this time there was neither paralysis nor salivation, so that these must have come on after his removal. It was a great omission not to have asked the learned Doctor whether he had ever seen a case of insanity in which they made their appearance without the administration of mercury? We do not say that, if conscious of paralysis, and even salivation, belonging to the characteristics of this malady, he was bound *to volunteer* such a piece of information; but his evidence would have pleased us much better if he had, and we think would have been quite consistent with his own character as to the official appointment he holds. There are few points in forensic medicine more interesting than the nature and the manner of evidence as given by medical witnesses; and when the interests of the Profession, which are those of humanity as well as of science, are at stake in the person of a brother member, we should expect that men of enlarged and cultivated minds—men of liberal sentiments, and just views of the dignity of their own situation, would put out of sight any little considerations of rivalry or personal dislike, and speak of each other with the respect that is, at least, becoming." 176.

"With regard to Dr. Sutherland, his testimony corroborated the opinion of Dr. Powell, as to the impropriety of the plan of treatment. But this gentleman seems to have been truly unfortunate in the grounds of his opinion. It would appear as if he formed it from the details afforded by a servant—and that a discarded one. In his cross examination, also, he admits exceptions enough to eat up his evidence in chief. It is with no small regret that we find ourselves compelled to animadvert upon the TESTIMONIAL deportment of men of some eminence; but it has been the fate of the *most eminent* occasionally to have given too much scope for animadversion on similar occasions; and we will seize the occasion to say to our younger brethren, that, to us, who are in the habit of looking at medical Practitioners in the situation of witnesses, with a scrutinising eye, they appear in a disadvantageous light when they allow most important and most manifest parts of the truth to be wrung from them by a cross-examination. We know that even professional men are summoned by one of the litigating parties with the view of favouring that side of the case; but such considerations ought to be the farthest from the candid and independent mind, especially when called upon for an opinion, and not for a statement of facts." 176.

---

2. *Royal College of Surgeons.* The College has lately promulgated the following bye-law, namely, that certificates of attendance at lectures on anatomy, physiology, the theory and practice of surgery, and of the performance of dissections, shall not be received by the Court, "*except from the appointed professors of anatomy and surgery in the Universities of Dublin, Edinburgh, Glasgow, and Aberdeen, or from persons teaching in a school, acknowledged by the medical establishment of one of the recognized hospitals, or from persons being physicians or surgeons to any of those hospitals.*"

This regulation has excited some sensation, and called forth, through certain channels of the press, much boisterous abuse and vociferous declamation. This mode of remonstrance is calculated, we conceive, to frustrate the object in view. For if the College rescind its regulation under a torrent of abuse, it will brand itself with the character of imbecility and timidity. We shall, therefore, make a few calm, and we hope impartial observations, on this bye-law.

It affects, or at least interests, three classes of the profession—the old established lecturers, most of whom are attached to hospitals—the *aspirants* at future lectureships—and the pupils themselves. We say nothing of those who have been recognized lecturers hitherto, though not belonging to any hospitals, as Mr. Brookes, Mr. Carpue, Mr. Mayo, &c. because it is quite impossible that the above regulation can operate, in an *ex post facto* manner upon them.

In respect to the first and second classes, we think it is abundantly evident, that if private teachers continue to multiply in the manner they have lately done, a period will arrive, when the old-established, or hospital schools of anatomy must fall—and that principally on account of the difficulty of procuring subjects for dissection. It is quite impossible that they shall be able to cope with ten or twenty times their number of private teachers, whose zeal and industry will snatch away every subject that can be procured by the resurrection men. The question, then, seems to be, is this *commonwealth* of anatomical and surgical tuition preferable to a *monopoly* of it in the hands of hospital surgeons? We much fear that considerable evil and inconvenience will attend either state, if it come to be unrestricted on the one hand, or exclusive on the other.

Although there can be no doubt that simple anatomy and physiology may be as well taught by a young private lecturer as by the most experienced surgeon, yet it must be allowed, that morbid anatomy, and more especially surgery, will be best taught by the hospital surgeon. We should therefore greatly regret the decay or embarrassment of the hospital schools in this or any other city of the empire, through the operation of *multiplied* private teachers. At the same time, we think that the law in question, which, if put in rigid force, must go far to annihilate private lectures in anatomy and surgery, is one of great severity, and which nothing but urgent necessity should call into operation. It certainly shuts the door on merit, unless that merit be conjoined with good fortune and strong friends. Nor do we think it will be of *ultimate* advantage to the hospital teachers themselves. No principle of decay is more certain or fatal than that which results from security of monopoly. To withdraw *competition* from any pursuit or undertaking, moral or physical, is to sap the foundation of human energy, and foster indolence. In fine, *monopoly* must ever prove an antidote or torpedo to *emulation*. The consequences are obvious.

The regulation in question will produce inconvenience, if not injury, to many of the third class—the students themselves. It confines their studies to certain prescribed channels, instead of giving them the range of the world to acquire knowledge, where and how they please. By the existing laws the student must be three years—or three winters, (which is tantamount,) in one or more of the abovementioned schools; whereas, we think that, considering the difficulties thrown in the way of practical anatomy in this country, one of those winters ought to be allowed on the Continent, where bodies are so readily procured and dissections so easily prosecuted.

It appears hard, also, that our large provincial hospitals, as those of Bath, Bristol, Liverpool, Leeds, Norwich, &c. should be excluded from the privilege of conferring hospital time for attendance on them, while that privilege is granted to Aberdeen, the scanty population of which can never supply much field for hospital practice. To this it is answered that, in Aberdeen, there is a regular anatomical chair, without which, attendance on an hospital would, it is said, be worse than useless. How far elementary anatomy is taught in any of the provincial hospitals we know not, and therefore cannot comment on the objection made to the want of an anatomical chair.

We have now set the question before our readers, in as impartial a manner as we can. We have no private interest or feeling in the result—and only wish that the good of medical science itself may be consulted by those who agitate the question. In the above reflections we do not hope to please either side—because we think that impossible, without embracing, exclusively, the views of one or other party. We consider it our duty to allay rather than excite hostile and angry feelings in the profession. Those who fan the flame of discord, on such occasions, have too often a *selfish* motive at the bottom.

3. *Nisbet* versus *Parsons.* To most of our readers the name of Nisbet will be familiar. Many a character has he sustained, or rather assumed, through life, and he is now, it seems, greatly in want of a *new* one. He accordingly tried to *recover* one, by law, in an action lately brought against Mr. Parsons of Camberwell. He did not succeed. He will probably take to his old trade of literary hack to the Booksellers, for whom he drudged many a year, with the motto on his back—

Sic vos non vobis fertis aratra boves.

It appears that a "Medical Board" had been established at Kennington, under the superintendence of Dr. alias *Surgeon* Nisbet and Co. his colleague being a half-pay captain. Under this Board was placed a wounded boy by his ignorant parents. But a gentleman (who was to pay the bill) thought proper to send the family surgeon (Mr. Parsons) to see if all was going on right. Mr. Parsons did not approve of the surgical treatment pursued by the Board, and pretty freely exposed their bungling practice. This enraged the head of the Board, and an action was brought against Mr. Parsons, evidently with the view of gaining notoriety with a verdict, of which he made sure. But, alas! the glorious uncertainty of the law! When the Doctor was called on to produce his diploma, as a surgeon, he could only produce the evidence of Dr. Walshman, which evidence went to prove that Dr. Walshman knew Dr. Nisbet for twenty years—but in what character Dr. W. was quite unable to say! The action, of course, fell to the ground, and the "Medical Board" of Kennington Common has probably, by this time, bowed to the same fate. Sic transit gloria mundi!

4. *Apothecaries' Act.* A trial came on at York lately, in which a surgeon-apothecary sued a family whom he had cured of the itch. The whole bill only amounted to five or six pounds. The plaintiff was proved to be a regular surgeon, and had been in practice for some years previous to 1814; but in that year he had embarked on board a vessel as surgeon. He was consequently *not practising on terra firma on the 1st day of August*, 1815. He returned after the act had passed, and it was in his subsequent practice that the subject of dispute arose. He was non-suited, because he was not actually practising on the identical day on which the act became law!! We consider this as a most disgraceful quibble, subversive of justice, and, in fact, contrary both to the letter and spirit of the act itself, which says—"in practice as an apothecary prior to, *or on* the said first day of August, 1815." If it had been "prior to *and* on the day," in question, it would have been a different thing.

